# Heat stress-induced NO enhanced perylenequinone biosynthesis of *Shiraia* sp. via calcium signaling pathway

**DOI:** 10.1007/s00253-024-13142-1

**Published:** 2024-05-03

**Authors:** Zhuanying Bao, Yunni Chen, Zhibin Zhang, Huilin Yang, Riming Yan, Du Zhu

**Affiliations:** 1 Key Laboratory of Protection and Utilization of Subtropic Plant Resources of Jiangxi Province, Jiangxi Normal University, Nanchang, 330022 China; 2 Key Lab of Bioprocess Engineering of Jiangxi Province, College of Life Sciences, Jiangxi Science and Technology Normal University, Nanchang, 330013 China

**Keywords:** *Shiraia* sp. Slf14(w), Heat stress, Perylenequinones, Nitric oxide, Calcium signaling pathway

## Abstract

**Abstract:**

Perylenequinones (PQs) are natural photosensitizing compounds used as photodynamic therapy, and heat stress (HS) is the main limiting factor of mycelial growth and secondary metabolism of fungi. This study aimed to unravel the impact of HS-induced Ca^2+^ and the calcium signaling pathway on PQ biosynthesis of *Shiraia* sp. Slf14(w). Meanwhile, the intricate interplay between HS-induced NO and Ca^2+^ and the calcium signaling pathway was investigated. The outcomes disclosed that Ca^2+^ and the calcium signaling pathway activated by HS could effectively enhance the production of PQs in *Shiraia* sp. Slf14(w). Further investigations elucidated the specific mechanism through which NO signaling molecules induced by HS act upon the Ca^2+^/CaM (calmodulin) signaling pathway, thus propelling PQ biosynthesis in *Shiraia* sp. Slf14(w). This was substantiated by decoding the downstream positioning of the CaM/CaN (calcineurin) pathway in relation to NO through comprehensive analyses encompassing transcript levels, enzyme assays, and the introduction of chemical agents. Concurrently, the engagement of Ca^2+^ and the calcium signaling pathway in heat shock signaling was also evidenced. The implications of our study underscore the pivotal role of HS-induced Ca^2+^ and the calcium signaling pathway, which not only participate in heat shock signal transduction but also play an instrumental role in promoting PQ biosynthesis. Consequently, our study not only enriches our comprehension of the mechanisms driving HS signaling transduction in fungi but also offers novel insights into the PQ synthesis paradigm within *Shiraia* sp. Slf14(w).

**Key points:**

*• The calcium signaling pathway was proposed to participate in PQ biosynthesis under HS.*

*• HS-induced NO was revealed to act upon the calcium signaling pathway for the first time.*

**Supplementary Information:**

The online version contains supplementary material available at 10.1007/s00253-024-13142-1.

## Introduction

Perylenequinones (PQs) represent a class of natural photosensitizing compounds characterized by a central structure of 4,9-dihydroxy-3,10-diphenyldione. They were originally isolated from the bamboo pathogenic fungi *Shiraia bambusicola* and *Hypocrella bambusae* (Bao et al. 2023; Khiralla et al. 2022; Mulrooey et al. 2012). PQs have held a historical presence in Chinese folk medicine for centuries and were recognized for their effectiveness in addressing various skin disorders, gastric ailments, and rheumatoid arthritis (Liang et al. 2009; Zhenjun and Lown 1990). Beyond their traditional medical using, PQs showcase natural photosensitizer properties, boasting remarkable photodynamic attributes coupled with low toxicity. These characteristics render them potent contenders in realms such as anti-microbial, anti-cancer, and anti-viral applications (Al Subeh et al. 2020; Xu et al. 2023; Yang et al. 2019). PQs not only hold promise within the pharmaceutical sector but also exhibit a diverse array of potential applications across agriculture, cosmetics, food, materials, and feed industries (Bao et al. 2023; Su et al. 2010, 2011). These multifaceted utilities further underscore the significance of PQs in various contexts.

At present, the limited yield from the submerged fermentation (SmF) of *Shiraia* spp. falls short of meeting market demands, posing a challenge to scaling up PQ production (Bao et al. 2023; Li et al. 2006; Liu et al. 2018a; Xu et al. 2023). Therefore, comprehending the biosynthetic pathway of PQs, streamlining production cycles, and enhancing production efficiency have emerged as pivotal factors in realizing PQ production through SmF (Bao et al. 2023). Over the recent years, a concerted effort from researchers has focused on identifying regulatory elements influencing the yield of *Shiraia* spp. during SmF. These factors encompass variables like light exposure, carbon and nitrogen sources, amino acids, inducers, temperature, and even genetic modifications aimed at achieving optimal PQ yield (Chen et al. 2022; Liu et al. 2018a; Xu et al. 2023; Yan et al. 2014; Yang et al. 2009). As a result, to achieve substantial gains in PQ production, the imperative task of delving into SmF techniques for *Shiraia* spp. remains a cornerstone pursuit.

In general, high temperatures can lead to heat stress (HS) which has been widely used in microbiology to study this effect (Luo et al. 2021; Wen et al. 2022). HS inhibits mycelial growth in a variety of fungi, and even damages cells and causes their death (Liu et al. 2018c; Qiu et al. 2018; Song et al. 2014; Xu et al. 2021). Faced with high-temperature environments, fungi usually mitigate the impact of HS by bolstering the accumulation of intracellular and extracellular secondary metabolites (Hu et al. 2022b), while also activating the expression of heat shock proteins (HSPs) (Xu et al. 2021; Zhang et al. 2019). Recent investigations have revealed elevated levels of HA (hypocrellin A) in *S. bambusicola* (GDMCC 60438) when the incubation temperature was increased from 26 to 32 °C (Wen et al. 2022). Additionally, our group found that *Shiraia* sp. Slf14(w) responds to HS by increasing the production of PQs (Xu et al. 2023). These above findings have prompted extensive explorations into the mechanisms fungi employ to cope with HS. It is noteworthy that specific signaling molecules in different fungal species have been implicated in orchestrating responses to HS. For instance, in *Pleuotus ostreatus*, the MAPK signaling pathway experiences a twofold amplification in the face of HS (Zou et al. 2018). Similar results were also reported in *Lentinula edodes* (Wang et al. 2018). Moreover, certain studies propose that NO assumes a signaling role in the synthesis of ganoderic acid (GA) induced by HS treatment, with a close association to Ca^2+^ (Liu et al. 2018b). Also, we revealed that HS-induced NO enhanced PQ biosynthesis of *Shiraia* sp. Slf14(w) (Xu et al. 2023). However, despite the wealth of studies highlighting the involvement of diverse signaling molecules in the fungal response to HS, their precise roles in the transmission of HS signals remain enigmatic. To enhance our comprehension of the regulatory mechanisms underlying fungal responses to HS, further research into other potential signaling systems involved in HS response is necessary.

Ca^2+^ serving as versatile secondary messengers, plays intricate roles in almost all abiotic stress responses (Falco et al. 2009; Dong et al. 2022; Zhu et al. 2019). For a particular abiotic stress, Ca^2+^ not only interacts with its perception but also ensures subsequent signal transduction across various functions (Dong et al. 2022). When cells sense stimuli, the dynamic changes in intracellular Ca^2+^ concentration are co-regulated by mechanisms involving both the influx and efflux of Ca^2+^. In animals, Ca^2+^ signaling actively participates in oocyte meiosis (Lu et al. 2021) and can regulate functions of thyroid cells, neurons, and endothelial cells (Leong et al. 2021, 2022). Meanwhile, in plants, the intricate mechanisms of Ca^2+^ decoding have undergone extensive scrutiny, encompassing the regulation of guard cell aperture, pollen tube growth, root hair development, and its interplay with nitrate responses (Brost et al. 2019; Pan et al. 2019; Wang et al. 2021; Zeb et al. 2020). In contrast to animals and plants, research on the physiological roles of Ca^2+^ in fungi has been relatively limited, but this realm has experienced notable growth in recent times. Studies indicate that Ca^2+^ is implicated in fungal hyphal growth, NO synthesis, and the biosynthesis of secondary metabolites (Liu et al. 2018a; Wang et al. 2012). Furthermore, mounting evidence points to an intimate link between the genesis of calcium signals and specialized calcium decoding systems. These systems can directly connect the latter to downstream targets, thereby instigating subsequent stress-related reactions (Zhu et al. 2013). Researchers have unearthed that the accumulation of H_2_O_2_ can act as a trigger for initiating calcium signal generation (Steinhorst and Kudla 2013). Another study posits that in plant roots, the kinetics and duration of NO_3_^−^ and calcium signal production show similarity, hinting at a potential correlation between calcium signaling in mesophyll cells and NO_3_^−^ responses (Liu et al. 2017). Moreover, investigations have demonstrated that NO and Ca^2+^ collaboratively enhance the synthesis of GA induced by HS (Liu et al. 2018b). Nonetheless, the precise interplay between the role of NO and the mechanisms triggering calcium signals remains a pivotal inquiry for forthcoming research endeavors.

In this study, we delved into the effects of HS-induced Ca^2+^ and the calcium signaling pathway on the biosynthesis of PQs. Furthermore, we explored the intricate relationship between HS-induced NO, Ca^2+^, and the calcium signaling pathway. This investigation not only expands our comprehension of the signaling mechanisms involved in HS responses in fungi but also lays the foundation for a novel approach to PQ biosynthesis within *Shiraia* sp. Slf14(w). By shedding light on the intricate interplay of these signaling pathways, our research contributes to the broader understanding of fungal responses to environmental stimuli and offers new avenues for optimizing the production of valuable secondary metabolites.

## Materials and methods

### Microorganism and culture conditions

The fungal strain *Shiraia* sp. Slf14(w) (CCTCC M209294) used is a naturally low PQ titer mutant of the endophytic fungus *Shiraia* sp. Slf14 (CCTCC M209294, the whole genome sequence number AXZN00000000) (Chen et al. 2022; Yang et al. 2014; Zhu et al. 2010). The fungal strains were stored in 20% glycerol tubes in a − 80 °C freezer.

The fungal medium used in this study was potato fructose medium (PFB) (200 g/L potato extract, 20 g/L fructose). The original seeds from a slant at 4 °C were cultured onto potato fructose agar (PFA) plates for a week at 28 °C to begin the liquid culture. Then, the mycelia were incubated for 3 d at 28 °C and 160 rpm in the light. Finally, each 250-mL conical flasks containing 70 mL of medium and 4 mL of homogeneous mycelium seed broth was placed in a shaker (Hu et al. 2012). The broth was cultured for 8 days as previously mentioned (Chen et al. 2022; Xu et al. 2023).

### Treatments with HS and chemical reagents

Fungal strains cultivated in PFB for 2 days were HS-treated from 0 to 8 h at 40 °C, respectively, and then recultured in a shaker at 28 °C (Xu et al. 2023). The amounts of gene transcripts or the cytoplasmic concentrations of NO and Ca^2+^ were measured at various culture periods. After 8 days of culture, the strains were collected, and fungal biomass, carbon source utilization, and PQ biosynthesis of *Shiraia* sp. Slf14(w) with and without HS treatment were analyzed. Prior to HS treatment, the strains were exposed to the relevant chemical reagents at specific concentrations for at least 30 min. Chemical reagents include sodium nitroprusside dihydrate (SNP), *N*ω-nitro-l-arginine, 2-(4-carboxyphenyl)-4,4,5,5-tetramethylimidazoline-1-oxyl-3-oxide potassium salt (cPTIO), and NS-2028, the calcium signaling pathway inhibitors such as ethylene glycol-bis(2-aminoethylether)-*N*,*N*,*N*′,*N*′-tetraacetic acid (EGTA), LaCl_3_, neomycin, chlorpromazine, tacrolimus (fk506), and cyclosporine A (CsA). CaCl_2_ and SNP were added in the experiments where exogenous calcium and SNP were needed to be provided following EGTA or cPTIO pretreatment for 30 min.

### Analysis of biomass growth and residual sugars

Three parallel samples were taken each time. Averages were calculated from three consecutive experiments. Dry cell weight (DCW) analysis was used to quantify biomass accumulation. The mycelia grown for 8 days were screened with a 200 mesh screen, cleaned three times with ultrapure water, drained with a vacuum pump, and then dried in a 50 °C oven to a constant weight. Residual sugar in fermentation broth was determined using the 3,5-dinitrosalicylic acid (DNS) method (Breuil et al. 1985).

### Extraction and analysis of intracellular and extracellular PQs

The PQs were extracted as described by Liu et al. (2018a). The dried mycelia were weighed and ground in a crusher. Then, 1.0 g dried mycelia were wrapped into cuboids with filter papers to extract pigments by Soxhlet extraction. The reflux liquid was evaporated at 40 °C and dissolved in 10 mL acetonitrile. Meanwhile, the 20 mL fermentation broth was extracted with dichloromethane until the fermentation broth was colorless. Then, the pigments were collected by rotary evaporation at 40 °C and dissolved in 10 mL acetonitrile. The collected pigments were filtered with 0.22 µm-nylon filter for determining intracellular and extracellular PQ content, respectively. According to Tong et al. (2017), individual PQs were measured by high performance liquid chromatography (HPLC, Waters 2996 system, Milford, MA, USA). HPLC was equipped with a YMC-Triart C18 column (250 mm × 4.6 mm, 5 µm, YMC Co. Ltd., Tokyo, Japan); the mobile phase was 70% acetonitrile and 30% water; a flow rate was 1.0 mL/min, and the monitoring wavelength was 460 nm. The total PQs is the sum of all individual PQs (Liu et al. 2018a; Xu et al. 2023).

### qRT-PCR analysis of gene expression

Different mRNA expression levels of *Shiraia* sp. Slf14(w) in response to HS were detected by qRT-PCR analysis. The mycelia were cultured for 2 days and treated with HS at 40 °C for 8 h. Three parallel samples of mycelia were taken at different times during and after the HS treatment, respectively. The mycelia were washed 3 times with sterile ultrapure water and then frozen in liquid nitrogen about 1 h for RNA extraction. The total RNA was extracted using the TRIzol reagent (Invitrogen, Carlsbad, CA, USA). The residual genomic DNA was then digested with RNA-free DNase I (TaKaRa, Dalian, China). RT-PCR was performed on ABI 7500 real-time PCR system (Applied Biosystems, Foster City, CA, USA) using SYBR Premix ExTaqTM (TaKaRa, Dalian, China) for reverse transcription. Amplified GAPDH transcripts were used as an endogenous control (Supplemental Table S1) because its expression level was relatively stable. PCR conditions: pretreatment at 95 °C for 10 min, denaturation at 95 °C for 10 s, annealing at 55 °C for 30 s, extension at 60 °C for 34 s. Then, 40 cycles of amplification were performed (95 °C for 10 s, 55 °C for 30 s, 60 °C for 34 s). The relative expression of genes was determined by the efficiency-corrected ΔΔCt method and the difference in the number of cycles in the linear amplification stage between samples (Livak and Schmittgen 2001). Primer pairs were designed using Primer 5 (Premier Biosoft, http://www.premierbiosoft.com/primerdesign/oligo/oligo.html). The gene fragments were amplified by real-time PCR using the primers are shown in Supplemental Table S1.

### Determination of intracellular Ca^2+^ by fluorescent probe

Intracellular Ca^2+^ level was measured by Ca^2+^ fluorescent probe Fluo-3/AM (Beyotime, Shanghai, China). Fluo-3/AM is a fluorescent dye that can penetrate the cell membrane, and it can be cut by some lipases in the cell to form Fluo-3, which binds Ca^2+^ to produce strong fluorescence (Hayashi et al. 1992; Rijkers et al. 1990). First, a suitable amount of mycelium was picked from the culture medium and placed on glass slides, and 5 µM Fluo-3/AM fluorescent solution was added to completely cover the mycelia. Then, the slides were incubated in dark at 28 °C for 60 min to introduce the fluorescent probe into the cells and rinsed with deionized water for three times. Next, the washed slides were incubated in dark at 28 °C for 30 min and observed under an inverted fluorescence microscope Eclipse Ti-s (Nikon, Tokyo, Japan). The fluorescence intensity was statistically analyzed by ImageJ software (NIH, Bethesda, MD, USA).

### Statistical analysis

All experimental data shown in the study were from three separate samples to ensure that the trends and relationships observed in the experiments are reproducible. The error bars in the graphs represent the standard deviation (SD) from the triple mean. *T*-tests were used to analyze the two comparison samples. Multiple comparisons were made using Duncan’s Multiple Difference Test. *P*-values < 0.05 were considered statistically significant.

## Results

### qRT-PCR analysis of related genes of the calcium signaling pathway and PQ biosynthesis under HS treatment

**Fig. 1:**
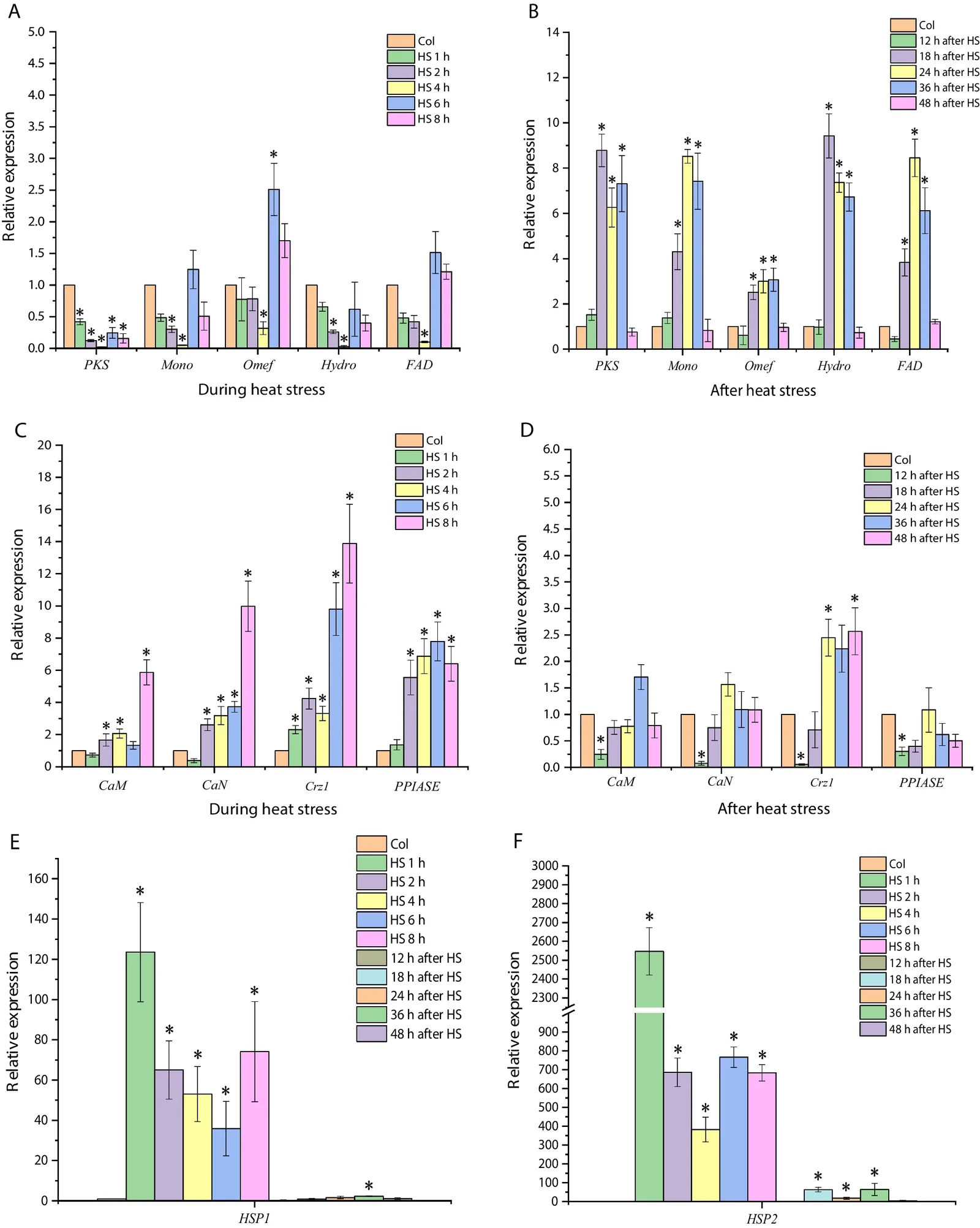
The relative transcription levels of target genes in heat stress treatment group and control group were detected by real-time fluorescence quantitative PCR. Asterisk (*) represents a significant difference compared with the control group (*P* < 0.05). Col and HS represent the control group cultured at 28 °C and HS group at 40 °C, respectively. CaM, calmodulin; CaN, calcineurin; Crz1, calcineurin-responsive zinc finger transcription factor; DCW, dry cell weight; FAD, FAD/FMN-dependent oxidoreductase; Hydro, hydroxylase; HS, heat stress; HSP, heat shock protein; Mono, FAD-dependent monooxygenase; Omef, *O*-methyltransferase; PKS, polyketide synthase; PPIase, prolyl cis–trans isomerase; PQs, perylenequinones

In a previous study, we found that HS treatment promoted the production of PQs, and the total PQ yield reached 577 ± 34.56 mg/L, which was 20.89-fold higher than that of the control. Also, the results indicated that HS-induced NO participated in the biosynthetic regulation of PQs (Xu et al. 2023). However, many studies have indicated that Ca^2+^ can participate in the stress response as a messenger in the process of an organism responding to various stresses including HS, and Ca^2+^-related signaling plays an important role in stress resistance as well as physiological and metabolic aspects of organisms (Liu et al. 2018c). Therefore, by using qRT-PCR, the expression of the most typical key proteins of the calcium signaling pathway such as calmodulin (CaM), calcineurin (CaN), and calcineurin-responsive zinc finger transcription factor (Crzl) in stress response was analyzed; the gene of prolyl cis–trans isomerase (*PPIase*) was also detected by qRT-PCR, since its product may interact with calcium-regulated phosphatases and may also be involved in the process of microbial response to HS (Mouhoumed et al. 2020). Moreover, the expression of PQ biosynthesis-related genes (*PKS*, *Mon*, *Omef*, *Hydro*, and *FAD*) was detected (Fig. 1).

The results indicated that the transcript levels of *CaM*, *CaN*, *Crz1*, and *PPIase* were significantly up-regulated during HS treatment, and especially, the transcript level of *Crz1* increased more than 14-fold at 8 h (Fig. 1C). After HS treatment, the transcript levels of *CaM*, *CaN*, and *PPIase* significantly decreased when strain Slf14(w) was re-cultured at 28 °C for 12 h, and then gradually restored to a level comparable to the control. The expression of *Crz1* also rapidly reduced in strain Slf14(w) re-cultured at 28 °C for 12 h, but then increased to twice of the control within 48 h after HS treatment (Fig. 1D). These results indicated that calcium signaling was also involved in the HS response of *Shiraia* sp. Slf14(w).

Further, the results showed that the expression levels of PQ biosynthesis gene clusters (*PKS*, *Mon, Omef*, *Hydro*, and *FAD*) were significantly down-regulated in the strain during HS treatment compared to the control (Fig. 1A). After HS treatment, all detected genes for PQ biosynthesis were significantly up-regulated in strain Slf14(w) re-cultured at 28 °C for 36 h, which could reach more than eightfold of the control (Fig. 1B). The above results suggested that HS activated the expression of key enzyme genes of the PQ biosynthesis pathway and thereby increased the production of PQs. In addition, when the strain was subjected to HS treatment at 40 °C, the genes for HSPs responded rapidly, and the transcript levels of *HSP1* and *HSP2* reached up to 120- and thousands-fold of the control during HS treatment, respectively (Fig. 1E, F). After returning to 28 °C of incubation, the expression of both *HSP1* and *HSP2* was decreased dramatically and recovered to the comparable level of the control (Fig. 1E, F). Based on these results, it can be inferred that the expression of PQ biosynthesis genes under HS treatment may be related to the calcium signaling pathway.

### HS regulates fungal growth and PQ biosynthesis via cytosolic Ca^2+^

Ca^2+^ is widely involved in the response to various biotic and abiotic stresses in organisms and is also an important signaling molecule. Our previous study demonstrated the involvement of Ca^2+^ in the PQ synthesis regulation in *Shiraia* sp. Slf14(w) (Liu et al. 2018a). In order to investigate whether Ca^2+^ was involved in the regulation of PQ synthesis under HS treatment, the Ca^2+^ chelator EGTA, the phospholipase C inhibitor neomycin, and the Ca^2+^ channel deterrent lanthanum chloride (LaCl_3_) were added in broths, and then subjected to HS treatment.

**Fig. 2:**
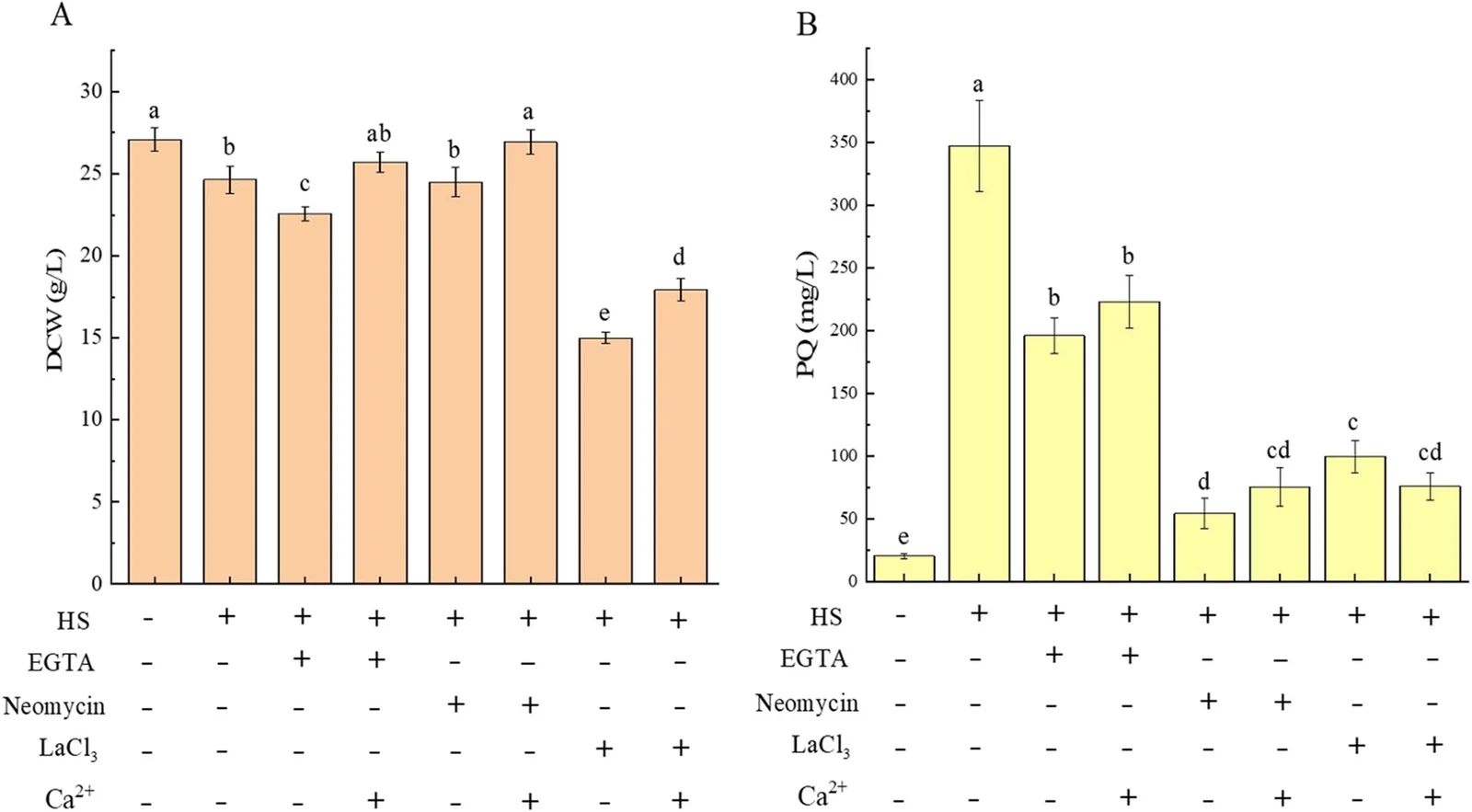
Effects of EGTA, neomycin, LaCl_3_, and Ca^2+^ on biomass (**A**) and PQ yield (**B**) of *Shiraia* sp. Slf14(w). The addition amounts of EGTA, neomycin, LaCl_3_, and Ca^2+^ were 5 mmol/L, 1 mmol/L, 5 mmol/L, and 0.6 g/L, respectively. Three independent replicates were set, and different letters indicated significant difference between treatments (*P* < 0.05). PQ, perylenequinone

The results indicated that the addition of neomycin (a phospholipase C inhibitor) had no significant effect on fungal biomass added into the broths, while the addition of EGTA (a putative extracellular Ca^2+^ chelator) decreased fungal biomass, and LaCl_3_ (a putative plasma membrane Ca^2+^ channel blocker) significantly inhibited fungal growth with a 39.13% decrease of the biomass (Fig. 2A), compared to the control which was only heat-stressed. The addition of Ca^2+^ before HS treatment promoted fungal growth, while the addition of Ca^2+^ inhibitor together with Ca^2+^ would appropriately mitigate the influence caused by the inhibitor on the growth of the fungus (Fig. 2A). This suggests that HS-induced Ca^2+^ enhances the heat tolerance of *Shiraia* sp. Slf14(w) and is involved in fungal growth regulation. For PQs, the addition of these above inhibitory compounds led to a decrease of HS-induced PQ yield in the fungal strain Slf14(w). EGTA treatment reduced the PQ content by 43.48%. Similarly, the treatment with neomycin and LaCl_3_ reduced fungal PQ production by 71.21% and 84.37%, respectively (Fig. 2B). Compared with EGTA addition, the inhibitory effects of adding neomycin and LaCl_3_ on PQ biosynthesis were more pronounced, probably because neomycin and LaCl_3_ are channel inhibitors that block the entry of Ca^2+^ from the extracellular to the intracellular compartment, while EGTA only chelates extracellular Ca^2+^. Taken together, these above data suggest that HS-induced intracellular Ca^2+^ is involved in the regulation of fungal growth and PQ biosynthesis. Moreover, the reduction of PQ production by these compounds also implies that different calcium sources are involved in the elevation of calcium levels in *Shiraia* sp. Slf14(w) under HS treatment.

### The calcium signaling pathway involved in the regulation of growth and PQ biosynthesis of HS-treated fungal strains

**Fig. 3:**
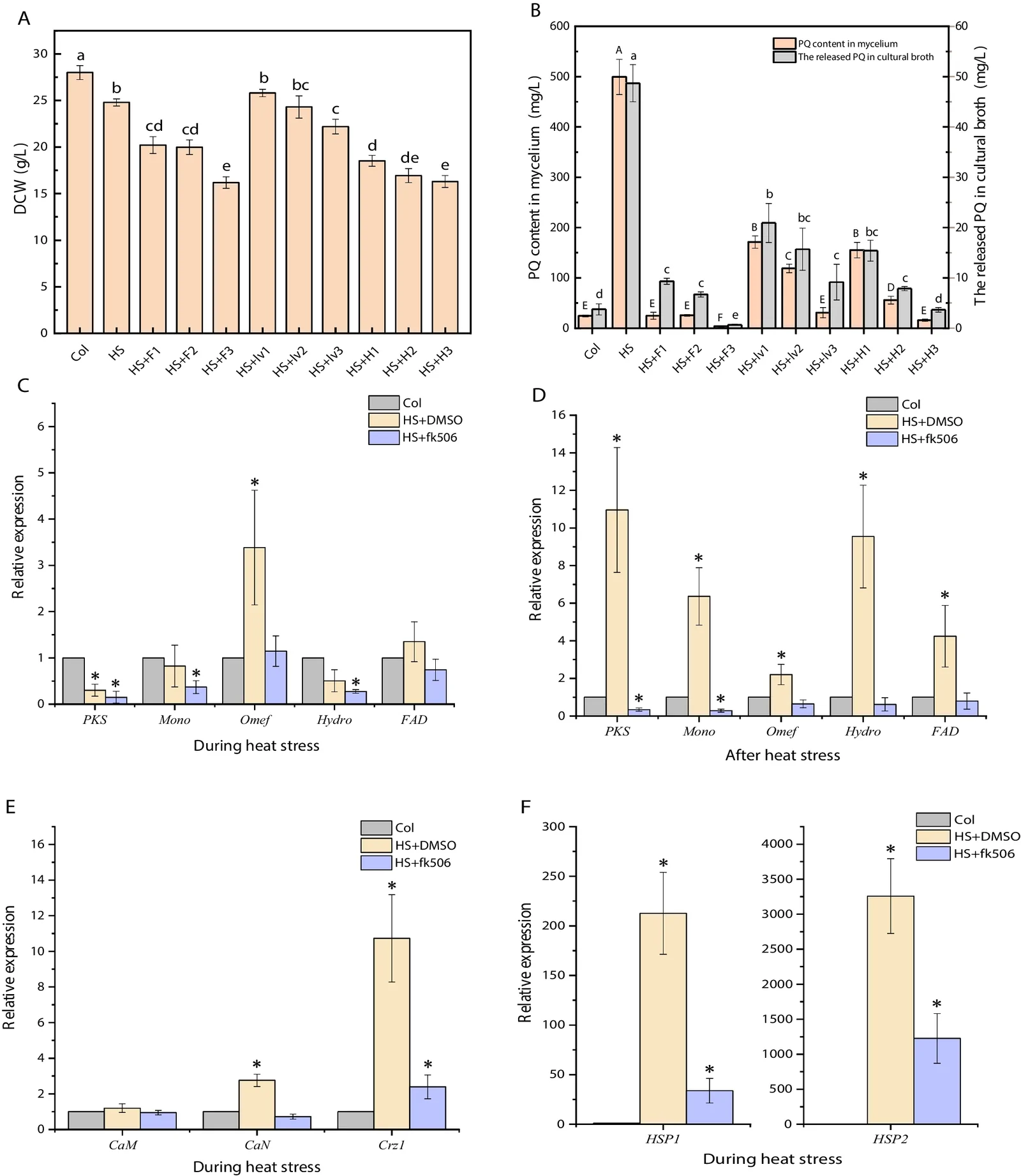
Effects of calcium signaling inhibitors on *Shiraia* sp. Slf14(w) biomass, PQ production, and related gene transcription. Effects of calcium signaling pathway inhibitors on biomass (**A**) and PQ production (**B**) of *Shiraia* sp. Slf14(w); effect of addition of tacrolimus (fk506) on transcriptions of PQ synthesis gene clusters (**C**), *CaM*, *CaN*, and *Crz1* in calcium signaling pathway (**E**) of strains during HS; effect of calcineurin inhibitor tacrolimus (fk506) on transcriptions of PQ synthesis gene clusters (**D**) and *HSPs* (**F**) in strain after HS. Col represents control group, HS represents heat stress; F1, F2, and F3 represent calcineurin inhibitor tacrolimus (fk506) at concentrations of 5 µM, 10 µM, and 50 µM, respectively; lv1, lv2, and lv3 represent calcineurin inhibitor chlorpromazine at concentrations of 0.5 mM, 1 mM, and 2 mM, respectively; H1, H2, and H3 represent the calcineurin inhibitor cyclosporine A at concentrations of 5 µM, 10 µM, and 20 µM, respectively; DMSO is the solvent of tacrolimus; fk506 is short for tacrolimus. CaM, calmodulin; CaN, calcineurin; Crz1, calcineurin-responsive zinc finger transcription factor; DCW, dry cell weight; FAD, FAD/FMN-dependent oxidoreductase; Hydro, hydroxylase; HS, heat stress; HSPs, heat shock proteins; Mono, FAD-dependent monooxygenase; Omef, *O*-methyltransferase; PKS, polyketide synthase; PQ, perylenequinone. Three independent replicates were set, and different letters indicated significant difference between treatments (*P* < 0.05). Asterisk (*) represents a significant difference compared with the control group (*P* < 0.05)

The results showed that elements of the intracellular calcium signaling pathway such as calmodulin (CaM), calmodulin phosphatase (CaN), and the transcription factor Crzl were significantly up-regulated in *Shiraia* sp. Slf14(w) during HS treatment (Fig. 1C). To further investigate whether these calcium signaling pathway-related proteins are involved in the regulation of fungal growth and PQ biosynthesis during HS treatment, the chemical reagents with different concentrations, including the CaM inhibitor chlorpromazine, the CaN inhibitor tacrolimus (fk506), and cyclosporine A, were supplemented to the broths on the second day of fermentation culture, respectively. The results showed that the addition of tacrolimus (fk506) and cyclosporine A caused a further decrease in fungal biomass compared to the control with HS treatment only, whereas the addition of chlorpromazine (CaM inhibitor) showed no significant effect on the fungal biomass, suggesting that CaN may play a more important role in the growth and development of the fungus (Fig. 3A). Further studies showed that the addition of the above compounds resulted in a decrease in fungal PQ content compared to the control which was only the HS treatment, and the inhibitory effect was enhanced with increasing concentrations of the inhibitors. Among them, tacrolimus (fk506) showed the most significant inhibitory effect and almost completely suppressed the increase of PQs caused by HS treatment (Fig. 3B). The above results indicated that CaM/CaN was involved in the growth of fungi and the biosynthesis of PQs during the response to HS and played a crucial role.

To further investigate the role of the calcium signaling pathway in PQ accumulation, the effect of the calcium signaling pathway inhibitor (tacrolimus) on the transcript levels of relevant genes was further detected by qRT-PCR. Accordingly, five genes from the PQ biosynthesis gene cluster (*PKS*, *Mono*, *Omef*, *Hydro*, and *FAD*) and calcium signaling pathway-related genes (*CaM*, *CaN*, and *Crz1*) were selected for detection. The results showed that the expression of the PQ biosynthesis gene clusters was significantly lower during HS treatment in the presence of the inhibitor than that under HS treatment alone (Fig. 3C). In addition, the expression of *CaN* and the transcription factor gene *Crz1* was also significantly reduced (Fig. 3E). It is suggested that CaN and the Ca^2+^/CaM-dependent protein kinase pathway may mediate the biosynthesis of PQs under HS treatment. Meanwhile, the transcriptional expression of *HSPs* was also suppressed by the addition of tacrolimus (Fig. 3F). It indicated that CaN and the Ca^2+^/CaM-dependent protein kinase pathway may also mediate the expression of fungal *HSPs* under HS treatment. When the fungus was re-cultured at 28 °C for 20 h after HS treatment, the addition of tacrolimus significantly decreased the transcriptional expression of the PQ biosynthesis gene cluster, which further confirmed that the relevant genes in calcium signaling were involved in the regulation of the biosynthesis of PQs under HS (Fig. 3D). Based on the results of adding CaM and CaN inhibitors, it can be assumed that HS-induced Ca^2+^ is involved in the regulation of fungal growth and PQ biosynthesis in *Shiraia* sp. Slf14(w) under HS through the Ca^2+^/CaM-dependent protein kinase pathway.

### The relationship between NO and Ca^2+^ under HS treatment conditions

**Fig. 4:**
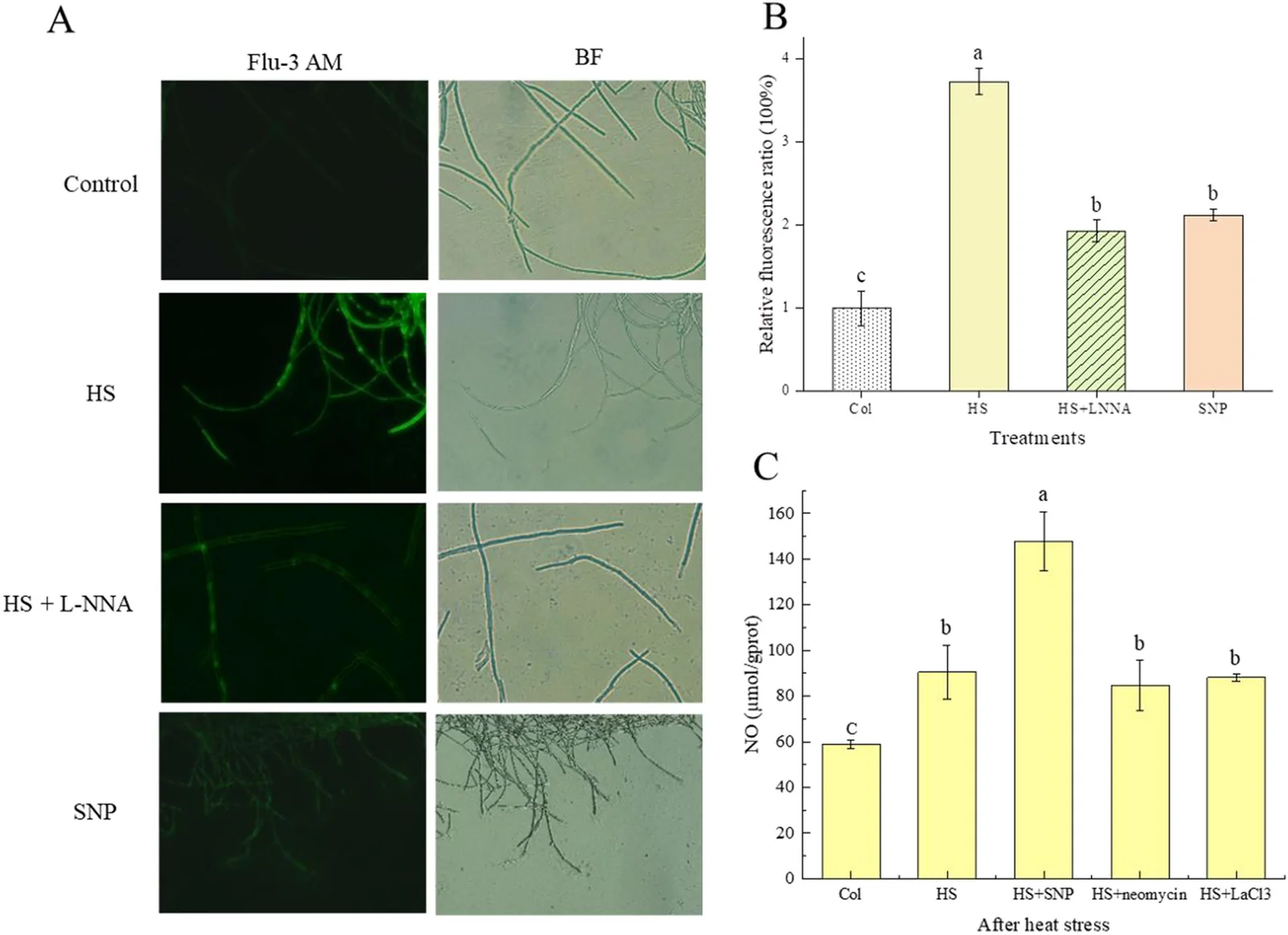
Effects of heat stress and NO on intracellular Ca^2+^ concentration in fungal cells. **A** L-NNA or SNP was added to the fermentation broth on day 2 of culture. After 30 min of treatment with L-NNA, it was transferred to 40 °C for 30 min of heat treatment. Then, the content of Ca^2+^ was detected by fluorescence intensity of Flu-3AM. **B** Variation of Ca^2+^ content under different treatment conditions. **C** The strain was subjected to HS treatment for 8 h after the addition of a Ca^2+^ inhibitor, and then returned to normal incubation for 20 h to determine the NO content in the strains. There were three independent replicates of the experiment, and letters indicated significant differences between treatments (*P* < 0.05). Flu-3 AM and BF stand for fluorescence light mode and normal light mode, respectively. HS, heat stress; LaCl_3_, lanthanum chloride; L-NNA, *N*ω-nitro-L-arginine; SNP, sodium nitroprusside

NO and Ca^2+^ have multiple pathways that are mutually regulated, which may influence each other in complex and ingenious ways. We previously reported that HS-induced NO, as a signaling molecule, triggered promoted PQ biosynthesis and efflux in *Shiraia* sp. (Xu et al. 2023). To better understand the Ca^2+^ signaling pathway, we observed the effect of NO on the intracellular calcium signaling pathway under heat-treated conditions. The calcium ion fluorescent probe Fluo-3AM was used to detect Ca^2+^ content. The results showed that HS caused a significant increase in intracellular Ca^2+^ concentration. However, pretreatment with a specific NO inhibitor L-NNA arrested up to 45% of HS-induced accumulation of Ca^2+^ compared to treatment with HS alone. The exogenous administration of SNP increased intracellular Ca^2+^ content by 51% compared with control (Fig. 4A, B). These data suggested that NO induced increased the intracellular Ca^2+^ concentration under HS treatment. Based on these results, it can be speculated that HS-induced NO could promote the increase of the intracellular Ca^2+^ concentration in *Shiraia* sp. Slf14(w).

NO and Ca^2+^ cross-talked with each other and they have an upstream and downstream relationship. To further investigate the relationship between NO and Ca^2+^ after HS treatment, SNP, neomycin, and LaCl_3_ were added before HS treatment, respectively. After HS, the strains were re-cultured at 28 °C for 20 h and the content of NO was determined. The results are shown in Fig. 4C. The NO content was significantly increased in the strain with the addition of SNP compared to that of HS treatment alone. Surprisingly, the content of NO in the strains with the addition of Ca^2+^ inhibitors (neomycin and LaCl_3_) was not significantly different from that of the strains treated with HS alone (Fig. 4C). The above experimental results indicated that inhibition of Ca^2+^ had no effect on NO production. Therefore, it can be inferred that Ca^2+^ may be located downstream of HS-induced NO.

### The relationship of NO on calcium signaling pathway under HS treatment conditions

**Fig. 5:**
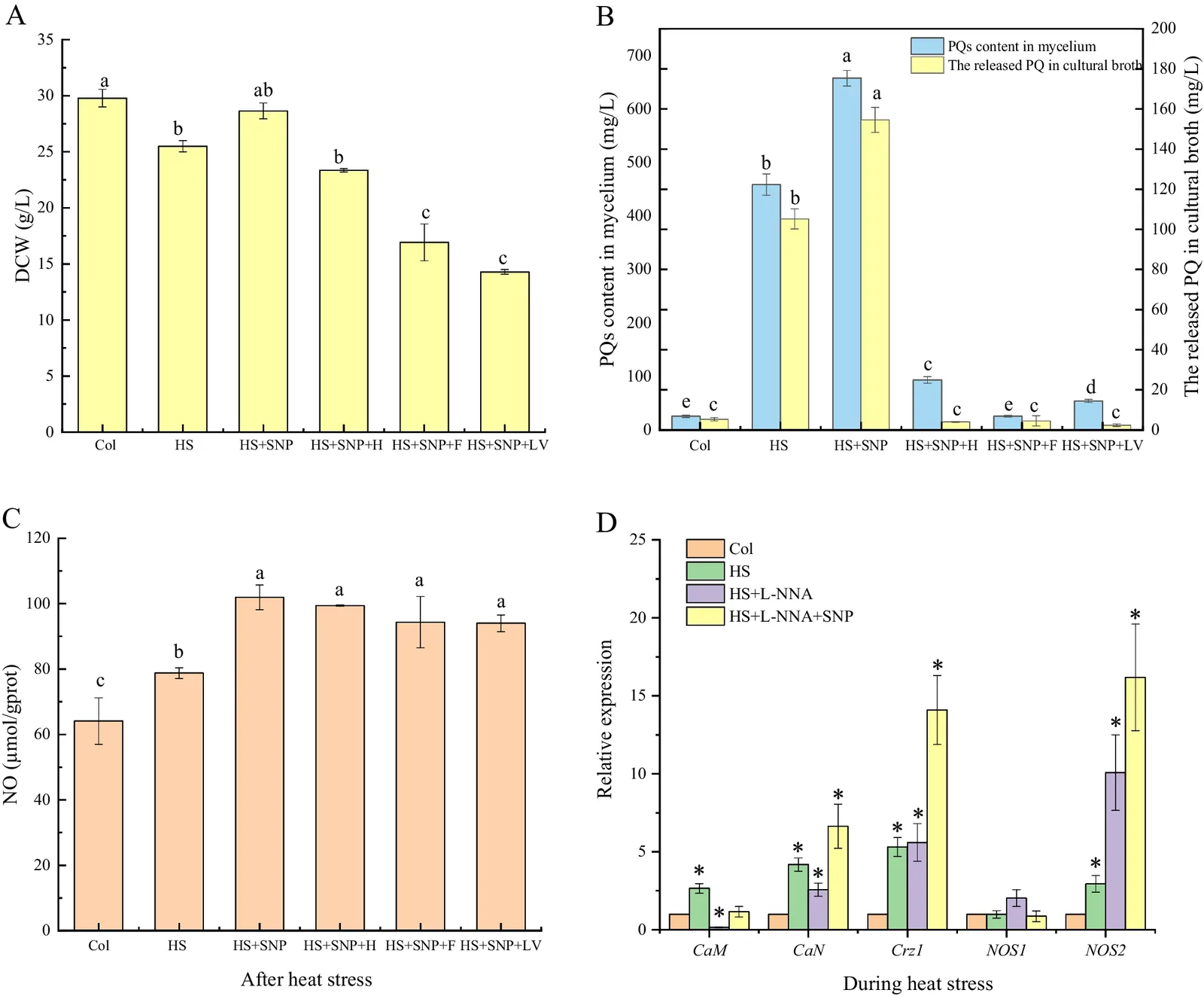
Study on the relationship between NO and calcium signaling pathway. Effects of adding NO donors and inhibitors of calcium signaling pathway before HS on biomass (**A**), intracellular and extracellular PQ contents (**B**), and NO content (**C**) in *Shiraia* sp. Slf14(w); effects of different treatments on *CaM*, *CaN*, *Crz1*, and *NOS* transcription levels (**D**). Col represents control group, HS represents heat stress, F represents tacrolimus at 10 µM (fk506), H represents cyclosporine A at 10 µM, and lv represents chlorpromazine at 1 mM. Three independent replicates were set, and different letters indicated significant difference between treatments (*P* < 0.05). Asterisk (*) represents a significant difference compared with the control group (*P* < 0.05). CaM, calmodulin; CaN, calcineurin; Crz1, calcineurin-responsive zinc finger transcription factor; NOS, nitric oxide synthase; PQs, perylenequinones

Calmodulin (CaM) is the main receptor for Ca^2+^ and plays a crucial role in the response to external factors (Zeng et al. 2015). To further investigate the relationship between NO and calcium signaling pathway under HS conditions, NO donors were added, followed by CaM inhibitors and CaN inhibitors prior to HS treatment. The addition of SNP improved the biomass of Slf14(w) compared to that of HS treatment alone. This suggested that SNP could relieve the damage caused by HS to the strain. In contrast, the biomass of the strain was significantly reduced when SNP was added followed by the addition of inhibitors (Fig. 5A). Based on the above data, it is indicated that inhibition of the calcium signaling pathway can inhibit the growth of *Shiraia* sp. Slf14(w). It shows that the calcium signaling pathway is essential for the growth of the fungal strain. In terms of PQ production, the addition of SNP could significantly increase the intracellular and extracellular PQ production and the PQ production reached 657.662 ± 14.538 mg/L, which was 25.5 times that of the control. The extracellular PQ production reached 154.596 ± 6.211 mg/L, which was 28.64 times that of the control (Fig. 5B). The inhibition of PQ production was observed when using chlorpromazine (CaM inhibitor), tacrolimus, and cyclosporine A (CaN inhibitor) in the presence of SNP. Among these inhibitors, chlorpromazine, a calmodulin inhibitor, showed the greatest effectiveness by reducing PQ production in the strain by 547.36 mg/L. This reduction was comparable to the level of PQs produced in the control group without any treatment (Fig. 5B). These results suggest that NO plays a role in the HS-induced calcium signaling pathway and PQ biosynthesis.

In order to further investigate the interaction between NO and the calcium signaling pathway in PQ biosynthesis under HS conditions, the strains cultured at 28 °C for 20 h after HS were collected and the NO content measured. The results showed that the strains with added SNP had significantly higher NO levels than those treated with HS only. Interestingly, the NO levels in the strains with added SNP followed by adding inhibitors of calcium signal transduction showed almost the same level as those with only SNP added (Fig. 5C). This suggests that the inhibition of the calcium signaling pathway does not affect the production of NO. Therefore, we speculate that the calcium signaling pathway plays a role downstream of HS-induced NO. To further validate the correlation between NO and the calcium signaling pathway during HS treatment, two sets of experiments were conducted. In one group, only the NOS (nitric oxide synthase) inhibitor L-NNA was used, while in the other group, both L-NNA and SNP were administered prior to HS treatment. Subsequently, the transcriptional expression of the calcium signaling pathway was assessed using qRT-PCR in both sets of experiments. Our findings revealed that the addition of L-NNA significantly reduced the expression of *CaM* and *CaN*, but increased the transcriptional level of NOS during HS treatment (Fig. 5D). The elevation in the *NOS* transcriptional level could be attributed to the inhibition of enzymatic NOS activity by L-NNA, which subsequently led to a decrease in NO content within the strains and consequently promoted *NOS* transcription. Conversely, in the experimental group where both L-NNA and SNP were added before HS treatment, the transcriptional expression of *CaM*, *CaN*, *Crz1*, and *NOS* exhibited a significant increase compared to the L-NNA group (Fig. 5D). This indicated that NO has a stimulatory effect on the expression of genes associated with the calcium signaling pathway under HS treatment. Therefore, the result further confirms our speculation that the calcium signaling pathway is downstream of HS-induced NO. In summary, the studies indicate that HS-induced NO enhances the transcriptional expression of gene clusters involved in PQ synthesis through the Ca^2+^/CaM-dependent protein kinase pathway, thereby increasing the production of PQs in *Shiraia* sp. Slf14(w).

## Discussion

As research into PQs deepens, their wide-ranging potential applications are gradually coming to light (Bao et al. 2023; Khiralla et al. 2022). However, these prospects are hindered by the limited natural stromal resources of *S. bambusicola* and *H. bambusae* (Wu et al. 1989; Tong et al. 2017). As a promising alternative technology for the production of PQs, the pursuit of SmF with *Shiraia* spp. has gained traction (Bao et al. 2023). Therefore, multiple factors influencing the fermentation production of PQs have been explored and optimized, including the addition of diverse nitrogen and carbon sources (Liu et al. 2020), gene editing techniques, incorporation of biotic or abiotic inducers (Du et al. 2013), mutation breeding, and controlled variations in light (Gao et al. 2018) and temperature conditions (Xu et al. 2023). Despite these efforts, meeting market demand remains elusive. Thus, in order to achieve cost-effective PQ production, it becomes imperative to further delve into enhancing PQ yields while concurrently investigating their biosynthetic pathway and regulatory mechanisms (Bao et al. 2023).

**Fig. 6:**
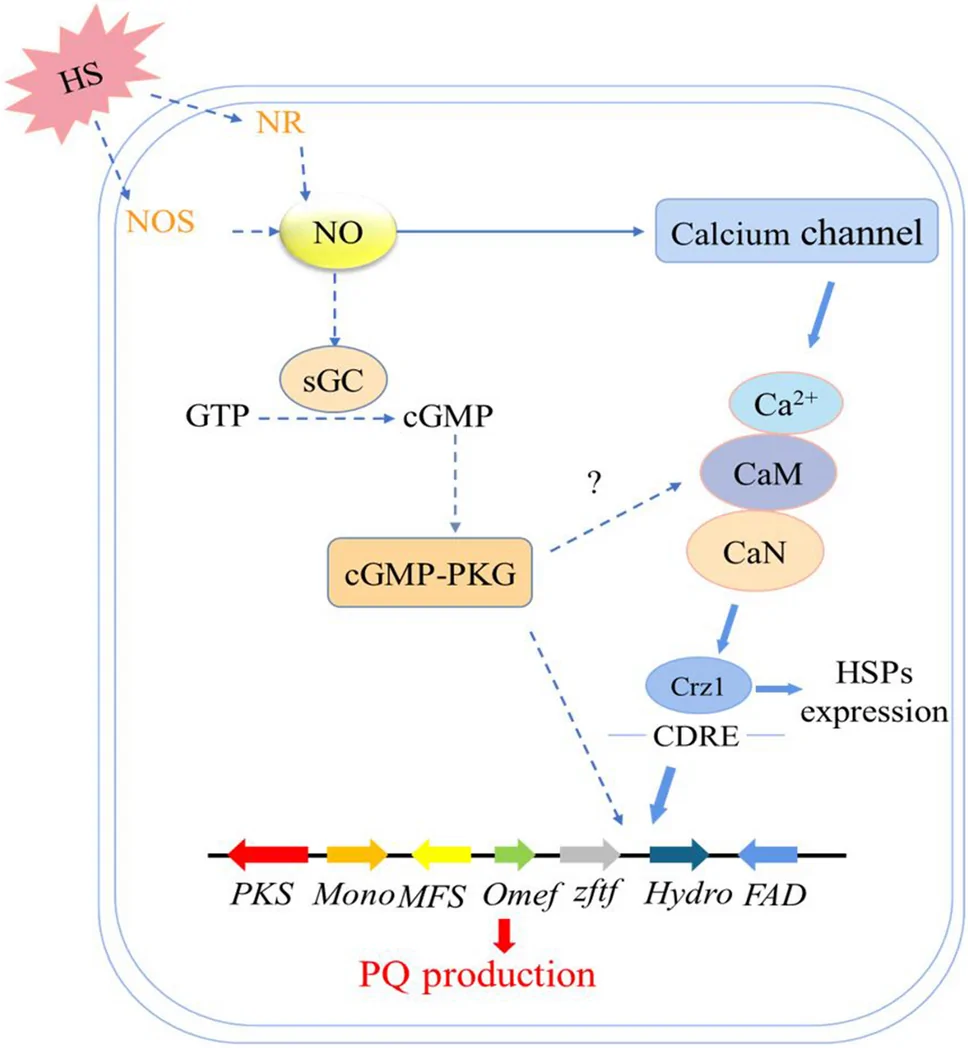
HS-induced NO regulates PQ synthesis through Ca^2+^/CaM-dependent protein kinase pathway. The blue solid arrows indicate data supported by our own experiments, the blue dotted arrows indicate data experimentally supported in other research, and question marks represent unknown events. CaM, calmodulin; CaN, calcineurin; Crz1, calcineurin-responsive zinc finger transcription factor; FAD, FAD/FMN-dependent oxidoreductase; HS, heat stress; HSPs, heat shock proteins; Hydro, hydroxylase; Mono, FAD-dependent monooxygenase; NOS, nitric oxide synthase; NR, nitrate reductase; Omef, *O*-methyltransferase; PKS, polyketide synthase; sGC, soluble guanylate cyclase; Zftf, zinc finger transcription

HS constitutes a critical environmental challenge that profoundly impacts microbial growth and development. Consequently, comprehending how microorganisms sense and respond to HS remains an essential endeavor. While extensive research has been conducted on HS, particularly in *Ganoderma lucidum*, not all the insights gleaned are universally applicable to fungi as a whole (Zhang et al. 2016). *Shiraia* sp. is particularly intriguing due to its capability to produce diverse secondary metabolites, with special attention on its synthesis of PQs. Earlier investigations have demonstrated that HS triggers a substantial increase in HA (hypocrellin A) content within *S. bambusicola* (GDMCC 60438), resulting in notable morphological shifts in mycelium when compared to lower temperatures (Wen et al. 2022). Generally, HS affects microbial growth, physiological metabolism, and biosynthesis of secondary metabolites. For instance, HS can reduce the viability of the ectomycorrhizal fungus *Tuber borchii* on host roots (Leonardi et al. 2017). HS down-regulated various proteins associated with metabolism of *P. ostreatus*, potentially reduced glycolysis and tricarboxylic acid cycle (TCA) intermediate metabolites, thereby impeded the growth and development of fungal strain (Zhao et al. 2018). To maintain energy equilibrium, cells must generate more energy, triggering pathways such as the glycolytic pathway (EMP), TCA, and the pentose phosphate pathway (PPP) in response to HS (Tan et al. 2018). In *G. lucidum*, HS inhibited mycelial growth and increased GA accumulation (Zhang et al. 2016). Our earlier research delved into the impact of HS treatment at 40 °C for 8 h on PQ biosynthesis in *Shiraia* sp. Slf14(w). The outcomes were striking, with a total PQ content reaching 577 ± 34.56 mg/L, marking a remarkable 19.89-fold increase compared to the control (Xu et al. 2023). Building upon this foundation, the present study sought to elucidate the mechanisms behind how HS amplifies PQ production. Our investigation led us to uncover that the heightened production of PQs under HS is a consequence of the synergistic interplay between the calcium signaling pathway and NO. Importantly, this study represents the pioneering instance demonstrating that HS-induced NO fosters the transcriptional expression of PQ biosynthesis gene clusters through the Ca^2+^/CaM-dependent protein kinase pathway. This intricate cascade effectively propels the augmentation of PQ production in *Shiraia* sp. Slf14(w) (Fig. 6).

Temperature-mediated regulation of fungal metabolites is intricately linked to gene expression modulation through activation of intracellular signaling pathways that stimulate the biosynthesis of secondary metabolites (Du et al. 2015). As a pivotal signaling molecule, NO has gained prominence intricately involved in the regulation of secondary metabolite production within both plant and fungal cultures (Zhang et al. 2012; Zhao et al. 2020). Studies have showcased that NO holds the potential to safeguard the mycelium of *Trichoderma harzianum* LTR-2 against HS while concurrently mitigating oxidative damage. This protective effect is achieved through the enhancement of nitric oxide synthase (NOS) and nitrate reductase (NR) activities (Yu et al. 2015). Additionally, NO emerges as a crucial player in countering HS-induced oxidative damage in the mycelium of edible mushrooms, effectively safeguarding their well-being (Kong et al. 2012). In a parallel vein, NO assumes a paramount role as a signaling molecule, intricately contributing to the metabolic regulation of growth and PQ biosynthesis within *Shiraia* sp. Slf14(w) (Xu et al. 2023). In our previous study, we observed a significant elevation in intracellular NO concentration upon exposure to HS, closely followed by marked increases in both intracellular and extracellular PQ concentrations. Our investigation included the use of specific inhibitors and scavengers, namely the NO scavenger cPTIO, NOS inhibitor L-NNA, and NR inhibitor Na_2_WO_4_, post-HS treatment. The outcomes of these interventions demonstrated that reducing NO content through these agents also led to a subsequent decline in PQ content. This cascade of events reinforces the role of heat stress-induced NO as a vital contributor to the synthesis and regulation of PQs in *Shiraia* sp. Slf14(w). In the context of fungal HS, although NO is considered to be a specific signaling molecule, it has also been shown that the production of Ca^2+^ by fungi such as *G. lucidum* and *P. ostreatus* can also act as a signaling molecule, not only to alleviate HS-induced stress, but also to promote the accumulation of secondary metabolites (Zhang et al. 2016). The elevation in fluidity of the plasma membrane, triggered by HS, precipitates the transient activation of specific calcium channels, resulting in the influx of Ca^2+^ into cells. This influx extends to mitochondria, potentially accompanied by inner mitochondrial membrane hyperpolarization and heightened ROS (reactive oxygen species) production. This interplay, in turn, orchestrates the direct or indirect regulation of ROS production and HSP synthesis through respiratory burst homologous proteins (Liu et al. 2018c; Pathak and Trebak 2018). At this, we found that the transcript levels of calcium signaling pathway-related genes were significantly up-regulated under HS treatment, and the transcript levels of the gene clusters related to the synthesis of PQs (*PKS*, *Mon*, *Omef*, *Hydro*, and *FAD*) were also significantly up-regulated (Fig. 1B). By adding Ca^2+^ and the calcium signaling pathway inhibitors, the production of PQs was consequently inhibited (Figs. 2B, 3BB). Further, we added NO donor SNP, NOS inhibitor L-NNA, and detected Ca^2+^ content. The results demonstrated that inhibition of the synergistic effect of Ca^2+^ and NO under HS treatment regulated the biosynthesis of PQs in *Shiraia* sp. Slf14(w) (Fig. 4).

Ca^2+^ acts as a second messenger, which links different input signals to many different specific responses. It is involved in the response to various biotic and abiotic stresses in organisms, such as light, drought, and high and low temperatures. The evolution of its concentration is related to the cellular perception of biotic stresses (Aldon et al. 2018). Under normal physiological conditions, intracellular free Ca^2+^ concentration is tightly and precisely controlled by the calcium signaling pathway complex. The calcium signaling pathway consists of various channels, sensors, and effectors located at the plasma membrane, intracellular storage organelles, and calcium buffering in the cytoplasm (Cyert and Philpott 2013). In unstimulated cells, the intracellular Ca^2+^ concentration is maintained at a 50–200 nm resting level, but the Ca^2+^ concentration in intracellular storage organelles is thousands of times higher. Upon stimulation, intracellular Ca^2+^ was released rapidly from the intracellular Ca^2+^ pools (Lauer et al. 2008; Yoshimoto et al. 2002). In this transient response, a variety of effectors are activated to adapt to environmental stimuli. The source of elevated intracellular Ca^2+^ is important for physiologic responses (Knight et al. 1997).

However, a small change in intracellular free Ca^2+^ rapidly translates into changes in the activity of several kinases, including Ca^2+^/CaM-dependent protein kinases involved in the regulation of many eukaryotic cell functions (Berridge et al. 2000). When intracellular Ca^2+^ levels are elevated, calmodulin neurophosphatase is activated by binding to the Ca^2+^/CaM complex. Calmodulin (CaM) is an acidic protein that acts as a major Ca^2+^ signaling receptor and can interpret important biological information encoded by changes in cytoplasmic Ca^2+^ concentration induced by environmental factors as well as the internal environment (Reddy 2001). CaM participates in the calcium signaling pathway by altering its interactions with various CaM-binding proteins. CaM is not catalytically active on its own, but by binding Ca^2+^, CaM can alter the activity of its binding proteins, thereby transforming local Ca^2+^ signals into specific physiological responses (Hartmann et al. 2014). Activated calcineurin dephosphorylates and activates the transcription factor Crz1. The Crzl transcription factor enters the nucleus and in turn activates a range of response genes by binding to calcineurin-dependent response elements. Studies have reported that Ca^2+^/CaM signaling regulates an array of plant defense processes. In tomato, in the response to insect pests, pest-induced ethylene signaling promotes the expression of *ERF15* and *ERF16*, and on the other hand, Ca^2+^/CaM signaling activates the transcriptional activity of *ERF16* through CaM2-ERF16 interactions, which in turn synergistically promotes the rapid accumulation of jasmonic acid (JA) by both ethylene and calcium signaling, thereby conferring the plant JA-mediated resistance to the insects (Hu et al. 2022a). The involvement of CaM and CaN in the regulation of mycelial growth and PQ synthesis was confirmed by the addition of the CaM inhibitor chlorpromazine, the CaN inhibitor tacrolimus, and cyclosporin A and played a crucial role under HS treatment (Fig. 3A, B). Our data showed that inhibition of Ca^2+^ and CaM/CaN activities effectively suppressed PQ production. It suggests that the HS-induced intracellular Ca^2+^ and CaM/CaN pathway are involved in the regulation of fungal growth and PQ biosynthesis, and CaN may be located at the key point of PQ biosynthesis signaling regulation. This further suggested that the Ca^2+^/CaM-dependent protein kinase pathway is an obligatory pathway for PQ biosynthesis.

Ca^2+^ functions as a signaling molecule in living organisms and its function is now well proven (Liu et al. 2018a; Zhang et al. 2016). There are growing evidences that Ca^2+^ and NO act together in pairs of organisms. In plant signaling, there are growing evidences that the two work together and complement each other. First, NO production is dependent on Ca^2+^/CaM (Stuehr and Griffith 1992). Second, NO is also one of the messengers that control Ca^2+^ homeostasis (Clementi and Meldolesi 1997). In addition, CaM may be regulated by NO at the post-translational level through *S*-nitrosylation (Jeandroz et al. 2013). In wheat, NO and calcium are effective in mitigating the adverse effects of salt stress, with NO being more actively involved in the antioxidant system and calcium being more favorable to ionic homeostasis (Tian et al. 2015). However, in microorganisms, fewer relevant studies have been reported on the relationship between signals, especially in fungi. Thus, it remains controversial which pathway is upstream of the other (Jeandroz et al. 2013). Our data showed that the addition of the NOS inhibitor L-NNA prior to HS treatment resulted in a decrease in intracellular Ca^2+^ concentration (Fig. 5D). In contrast, the addition of the NO donor SNP during incubation resulted in a significant enhancement of intracellular Ca^2+^ levels compared with the control, suggesting that NO can promote increased intracellular Ca^2+^ concentrations. In addition, after the addition of Ca^2+^ inhibitors followed by HS treatment, NO levels actually remained consistent with those of HS, suggesting that inhibition of the calcium signaling pathway had no effect on NO production (Fig. 5C). The above results indicated that Ca^2+^ acts downstream of HS-induced NO production. However, the addition of L-NNA during HS treatment significantly reduced the expression of CaM and CaN in the strain. On the contrary, in the experimental group where both L-NNA and SNP were added prior to HS treatment, the transcriptional expression of *CaM*, *CaN*, *Crz1*, and *NOS* was significantly increased compared with that in the L-NNA group. This indicated that NO could promote the expression of calcium signaling pathway-related genes during HS treatment. Accordingly, our results further demonstrated that the calcium signaling pathway is located downstream of HS-induced NO (Fig. 6). Therefore, this study provides the first evidence that both NO and Ca^2+^ co-regulate the biosynthesis of PQs in S*hiraia* sp*.* Slf14(w) under HS treatment.

Overall, our data confirm the existence of a novel signaling pathway to increase the content of PQs, in which NO and calcium can reinforce each other and work together to counteract HS. The combined application of exogenous NO and Ca^2+^ significantly increased the content of PQs under HS conditions. Interestingly, Ca^2+^ antagonized the toxic effects of exogenous NO. Thus, the combined application of NO and Ca^2+^ could compensate for each other’s deficiencies and achieve a synergistic effect in mitigating the adverse effects of heat stress. This finding enables us to elucidate the signaling mechanism of HS in *Shiraia* sp*.* Slf14(w) at a more advanced level, providing a new strategy for fungi to enhance the production of secondary metabolites.

## Supplementary information

Below is the link to the electronic supplementary material.

Supplementary file1 (PDF 73 KB)

## Data Availability

All data generated or analyzed during this study are included in this published article [and its supplementary information files].
