# Supplementary material for: Heat stress-induced NO enhanced perylenequinone biosynthesis of Shiraia sp. via calcium signaling pathway
Source: Appl Microbiol Biotechnol. 2024 May 3;108(1):317. doi: 10.1007/s00253-024-13142-1 (PMC11068690; doi:10.1007/s00253-024-13142-1)
Supplement: Supplementary file 1 — Supplementary file1 (PDF 73 KB) [file 253_2024_13142_MOESM1_ESM.pdf]

Applied Microbiology and Biotechnology

Heat stress-induced NO enhanced perylenequinone biosynthesis of *Shiraia* sp. via calcium signaling pathway

Zhuanying Bao[1,2#] · Yunni Chen[1#] · Zhibin Zhang[1] · Huilin Yang[1] · riming Yan[1] · Du Zhu[1,2*]

* Du Zhu zhudu12@163.com

# contributed equally to this work.

1 Key Laboratory of Protection and Utilization of Subtropic Plant Resources of Jiangxi Province, Jiangxi Normal University, Nanchang 330022, China

2 Key Lab of Bioprocess Engineering of Jiangxi Province, College of life sciences, Jiangxi Science and Technology Normal University, Nanchang 330013, China

Supplementary Materials

Table S1 Sequences of primer pairs used for qRT-PCR assay

Target gene	Gene code	Primer name	Primer sequence (5'-3')
CAM	evm. TU.scaffold46_1.44	CAM-F	GCACCATTGACTTCCCAGAG
		CAM-R	AGAGATGAAGCCGTTGTTGT
CAN	evm. TU.scaffold13_1.242	CAN-F	CCATCCTGAATACCTGCTCT
		CAN-R	CGCTTGAACTCGGTTGACTC
CRZ1	evm.TU.scaffold11_1.251	CRZl-F	CGGAGTCGCAATCGCATTC
		CRZ1-R	GCATCGTTAGGTGACAAGGAG
NR	evm.TU.scaffold23_2.7	NR-F	CATCAGCCAGACCACCCAGAC
		NR-R	GCCGAGGTACTCGAACTTTCC
Mono	evm.TU.scaffold40_1.64	Mono-F	CGAGACCCAAATGACGACTAT
		Mono-R	ACGAGACGGCACAGGAAG
Omef	evm.TU.scaffold40_1.66	Omef-F	GACTGGTCGGACAAGTATGC
		Omef-R	GGCTCTGGCACAATGACTTC
PKS	evm.TU.scaffold40_1.63	PKS-F	CCTCTGATGTTGACGG
		PKS-R	CTTGGAGATGTGCTGC
FAD	evm.TU..scaffold40_1.70	FAD-F	TGCCGTCTGCTGGTCTTG
		FAD-R	ACACCAACGCCACCTACT
Hydro	evm.TU.scaffold40_1.69	Hydro-F	CTGGGTCAATAACGAAAA
		Hydro-R	CAGGAATCAGCCGAAGTC
PPIase	evm. TU.scaffold1_2.60	PPIase-F	GACCCTACGGGTACGGGAAGA
		PPIase-R	CGCAAGCGTGATGAAGAACTG
HSP1	evm. TU.scaffold23_2.137	HSP1-F	TAGCAAGGACGCAGCACAGGA
		HSP1-R	GCGAAGTCGGCATAGACACCA
HSP2	evm. TU.scaffold9_1.327	HSP2-F	GTCACGAACATTTCCGAGAACA
		HSP2-R	GCTTATCCGATTGCTTGCTTAC
GAPDH	evm.TU.scaffold5_1.217	GAPDH-F	TTGACCTGACTGTCCGCATC
		GAPDH-R	TGAGACAAGCTTGACGAAGT
